# Plant growth and phenolic compounds in the rhizosphere soil of wild oat (*Avena fatua* L.)

**DOI:** 10.3389/fpls.2013.00509

**Published:** 2013-12-17

**Authors:** Anna Iannucci, Mariagiovanna Fragasso, Cristiano Platani, Roberto Papa

**Affiliations:** Consiglio per la Ricerca e la Sperimentazione in Agricoltura, Cereal Research CentreFoggia, Italy

**Keywords:** *Avena fatua* L., phenolic compounds, plant growth, rhizosphere soil, root exudates

## Abstract

The objectives of this study were to determine the pattern of dry matter (DM) accumulation and the evolution of phenolic compounds in the rhizosphere soil from tillering to the ripe seed stages of wild oat (*Avena fatua* L.), a widespread annual grassy weed. Plants were grown under controlled conditions and harvested 13 times during the growing season. At each harvest, shoot and root DM and phenolic compounds in the rhizosphere soil were determined. The maximum DM production (12.6 g/plant) was recorded at 122 days after sowing (DAS; kernel hard stage). The increase in total aerial DM with age coincided with reductions in the leaf/stem and source/sink ratios, and an increase in the shoot/root ratio. HPLC analysis shows production of seven phenolic compounds in the rhizosphere soil of wild oat, in order of their decreasing levels: syringic acid, vanillin, 4-hydroxybenzoic acid, syringaldehyde, ferulic acid, *p*-cumaric acid and vanillic acid. The seasonal distribution for the total phenolic compounds showed two peaks of maximum concentrations, at the stem elongation stage (0.71 μg/kg; 82 DAS) and at the heading stage (0.70 μg/kg; 98 DAS). Thus, wild oat roots exude allelopathic compounds, and the levels of these phenolics in the rhizosphere soil vary according to plant maturity.

## Introduction

Wild oat (*Avena fatua* L.) is considered to be among the worst weeds throughout the world, and it can cause serious competitive yield losses in grain crops. It is an annual grass that is difficult to eradicate because the seeds shatter before crop maturation. As a consequence, many of the seeds are ploughed into the soil, where they can lie dormant for one or more years. Furthermore, management of this weed has become particularly problematic, as it has developed resistance to many of the herbicides that were once available for its control (Heap, 2009). The success of this weed is attributed to its adaptation to different biological and ecological conditions. Due to its competitive nature, wild oat reduces the availability of nutrients for crops, thereby reducing the crop yield both qualitatively as well as quantitatively (Khan et al., 2006). However, as reported by Narwal et al. (2005), the negative interactions between weeds and crops might be due to a combination of competition and the underground plant–plant interaction known as allelopathy.

Allelopathy is defined as any positive or negative effects that one plant has over another through chemicals that escape into the environment through root exudation, leaching and volatilization, and through the decomposition of plant residues (Rice, 1984). Root exudation has long been viewed as a passive leaching process. However, recent studies have shown that roots can synthesize, accumulate, and actively secrete several compounds into the rhizosphere soil (Bertin et al., 2003; Bais et al., 2004; Prithiviraj et al., 2007). Therefore, the rhizosphere must be considered the main site for allelopathic interactions, and a number of phytotoxic compounds have been identified in plant root exudates (Kruse et al., 2000).

Allelochemicals can influence vital physiological processes, such as respiration, photosynthesis, cell division and elongation, membrane fluidity, protein biosynthesis, and the activities of many enzymes, and they might also affect tissue water status (Field et al., 2006). As reported by Schumacher et al. (1983), wild oat is involved in interactions with wheat (*Triticum aestivum* L.) plants through the production and release of phytotoxic substances, and in particular, of phenolic compounds. These compounds are a class of the most important and common plant allelochemicals in the ecosystem (Li et al., 2010). In soil, phenolics can occur in three forms: free, reversibly bound, and bound. Generally, the first two forms are considered important from the standpoint of allelopathy, allowing phenolics to accumulate in rhizosphere soils.

Soil microorganisms also have important roles in allelopathy, as they have the potential to modify these effects through the degradation of toxic compounds or through the production of toxic compounds (Inderjit, 2001). Furthermore, while moving through the soil, allelochemicals might undergo transformation, as various factors within the soil environment, such as the physical, chemical, biological and physicochemical properties of the soil, can influence the activities of allelochemicals. Due to these interactions, the role of the soil should not be ignored in studies of the allelopathic potential of a plant (Inderjit et al., 2010). Indeed, the isolation and identification of chemicals from donor plants with biological activity does not necessarily demonstrate that these compounds interfere in nature through allelopathy (Inderjit, 2000).

At present, the study of phytotoxins released by intact roots of living crop plants is considered one of the most promising approaches to exploit allelopathy in annual crops (Duke et al., 2005; Macías et al., 2007). However, the extent of allelopathy by a plant can vary with age, and part and type of cultivar being used (Batish et al., 2001). Therefore, knowledge of the development of a weed species, such as wild oat, is important for our understanding of the plant growth and the accumulation of allelochemicals in the rhizosphere soil, and for the development of appropriate management practices.

The objectives of the present study were (i) to evaluate the growth dynamics and partitioning of assimilates in the above-ground and below-ground plant organs of wild oat; (ii) to identify the phenolic compounds with potential phytotoxic effects in the rhizosphere soil, and determine whether their concentrations vary during plant growth; and (iii) to compare the dynamics of the plant growth and the concentrations of these phenolic compounds in the rhizosphere soil.

## Materials and methods

### Plant growth and soil sampling

Seeds from wild-type oat populations were collected from agricultural fields at the Cereal Research Center farm located in Foggia (Italy) (41°28′ N, 15°34′ E; 76 m a.s.l.) in 2011, and stored at 4°C. These wild oat seeds were surface sterilized for 10 min in a 1:10 (v/v) dilution of commercial hypochlorite bleach, and then rinsed several times with distilled water (Rao et al., 2006). Five sterilized seeds were placed in each pot (diameter, 18 cm; height, 14 cm), which contained 2 kg of a soil mixture (soil: sand: peat, 60:30:10; v/v). The soil was taken from the surface layer of a field (0–20 cm), and was an unsterilized loam soil (USDA classification system) with the following characteristics: 21% clay, 43% silt, 36% sand, pH 8 (in H_2_O), 15 mg/kg available P (Olsen method), 8.0 mg/kg exchangeable K (NH_4_Ac), and 21 g/kg organic matter (Walkey–Black method). Silica sand with a grain size from 0.4 to 0.1 mm was used. A commercial product containing peat-moss was used (Terravera® Universal mould).

After emergence, the seedlings were thinned to one per pot. The pots were placed in a growth chamber with daily light/dark and temperature cycles that were changed as a function of the plant growth. Thus, the growth conditions were regulated according to the mean values of the photoperiod and temperature of the plain environments of southern Italy. A light of 1000 μmol photons/m^2^/s PAR was used. A dose of 60 kg/ha of 18/46 fertilizer (18% elemental N, 46% P_2_O_5_, by weight) and 90 kg/ha NH_4_NO_3_ (26% elemental N) were applied at sowing and at plant tillering, respectively. No further nutrient solutions were used. The plants were regularly watered to 70% of the field capacity. The control treatment consisted of three pots without plants, stored under the same conditions. The plants and soils were sampled 13 times during the growing season, according to the defined growth stages from tillering (41 days after sowing; DAS) to ripening (122 DAS). At each sampling, the heights of the plants were measured, and then they were collected by pulling them from the soil in the pots, with all of the plant material manually removed from the pot. The roots were carefully separated and shaken gently to collect the root-zone, or rhizosphere, soil. Rhizosphere soil samples from the wild oat plants were mixed thoroughly, sieved (2-mm mesh) to remove any root tissue and oven-dried at 30°C, under vacuum. The soils were then stored in a cool, dark, dry environment until analyses. The plants were cleaned and weighed as the shoots (separated into leaves, stems and ears) and the roots. The dry weights were recorded after drying in a forced-draught oven at 60°C for 48 h.

The leaf/stem ratio (LSR), shoot/root ratio (sum of the aerial plant parts/root; SRR) and source/sink ratio (leaf/sum of the other plant parts; SSR) were calculated from the dry weights. The harvest index was calculated as the ratio of the grain mass to the total above-ground biomass. The aerial biomass relative growth rate (RGR) was determined separately for the 12 short-term (4–11 days) growth periods, according to the classical approach (Hunt et al., 2002). The average values of the long-term (4 months) growth periods were calculated as the mean values of the growth periods. Furthermore, the RGR and shoot elongation rate (SER) were estimated throughout the experimental period, according to the functional approach: as the slopes of the linear regressions of the natural logarithm of the shoot dry matter (DM) and of the non-transformed data of the shoot length vs. time, respectively (Hunt et al., 2002). Both linear regressions were highly significant (*P* ≤ 0.01), and no quadratic effects were observed (*P* > 0.05). Confidence intervals for regression coefficients were determined at the 5% level of probability. Shoot and root DM were correlated allometrically, as described by Blaikie and Mason (1990). The linear regression model used was ln (shoot) = *a* + *b* ln (root). The slope, *b*, is the ratio of the RGRs of the shoots and roots. If *b* > 1, the RGR of the shoots is greater than the RGR of the roots.

### Phenolic compounds

One hundred grams of each oven-dried soil sample was extracted with 300 ml methanol (agitation, 48 h at 25°C; centrifugation, 1200 × *g* for 15 min). Pure methanol, a polar solvent, was used to extract the free phenolic acids from the soil because of its high extraction efficiency for the hydrophilic compounds (Kong et al., 2006). Furthermore, the methanol has a protective role, because it can prevent phenolic compounds from being oxidized by enzymes, such as phenoloxidases (Proestos et al., 2006). The extracts were concentrated on a rotary evaporator (40°C), and the residues were dissolved in HPLC grade methanol. Then the samples were filtered through 0.45-μm filters prior to injection of 2 μl of each sample into the HPLC system (Agilent HP 1200). Analysis of the allelopathic compounds was repeated three times, with three extracts in each sample.

The HPLC diode array detector (Agilent, Waldbronn, Germany) was equipped with a reverse-phase Zorbax SB-C18 column (Eclipse 100 × 2.1 mm, 1.8 μm). The column oven temperature was set at 35°C. Linear gradient elution was used for the analysis, with the mobile phases of acetonitrile (solution A) and aqueous 1% acetic acid (solution B), as follows: 100% solvent B at 0 min; 85% solvent B at 12 min; 50% solvent B at 20 min; 0% solvent B at 22 min; 100% solvent B at 24 min; isocratic elution of 100% B, 24–30 min. The flow rate was 0.4 ml/min, with detection at 280 nm. The reference standard compounds were purchased from Aldrich (Aldrich Chemical Co., USA) and run alone and as mixtures: gallic acid, ferulic acid, *p*-coumaric acid, 4-hydroxybenzoic acid, vanillic acid, clorogenic acid, caffeic acid, trans-cinnamic acid, 3,4-hydroxybenzoic acid, syringic acid, salicylic acid, syringaldehyde and vanillin.

**Figure 1:**
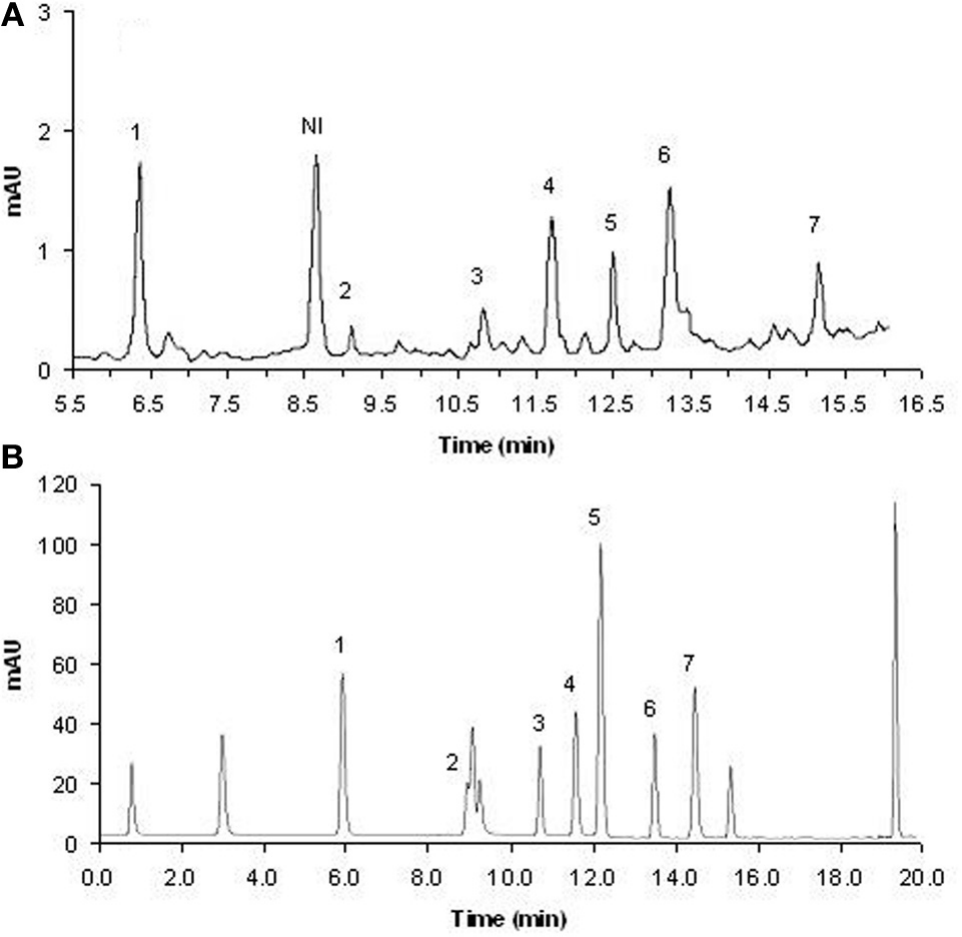
**Chromatographic profile of phenolic compounds in the rhizosphere soil of wild oat (A) and for standards (B).** 1, 4-hydroxybenzoic acid; 2, vanillic acid; 3, syringic acid; 4, vanillin; 5, *p*-cumaric acid; 6, syringaldehyde; 7, ferulic acid; NI, not identified.

Retention times of these standard compounds and their major peaks in the extract were recorded. Figure 1 shows a chromatogram corresponding to a rhizosphere soil sample of wild oat; the good resolution and separation of the chromatographic peaks corresponding to the absorption at 280 nm can be seen. Seven phenolic compounds (out of 13) were identified through their retention times and their UV spectra as compared to those of the standards, and their concentrations were calculated by interpolation of the peak areas on the chromatograms of the HPLCs to a standard curve constructed using the peak areas of the authentic phenolic acids. The linearity of calibration curves for all compounds was very good (*R*^2^ > 0.99). The detection limits (L_D_) and the recovery of phenolic compounds from the soil were estimated by adding measured amounts of each representative standard to the samples prior to extraction of soil samples. Spiked samples were extracted as previously described, but with six replicates. The standard deviation, S, of the measured concentrations was calculated for each compound, and the L_D_ was taken as 3S. L_D_ values ranged from 0.11 μg/ml for vanillin to 5.95 μg/ml for ferulic acid. The average recoveries of known amounts of allelochemicals added into the soil were 83.3% (ferulic acid), 96.2% (*p-coumaric* acid), 75.3% (4-hydroxybenzoic acid), 96.1% (vanillic acid), 85.1% (syringic acid), 64.7% (syringaldehyde) and 60.4% (vanillin), which were used to correct the concentrations determined. As reported by Cantor et al. (2011), due to the method used for drying the soil samples, some of the phenolic compounds might have undergone conversion, which would at least partially explain our recovery efficiencies of these phenolic acids.

In the present study, we report the net phenolic compounds concentrations, as obtained by subtracting the values from the control soil.

### Statistical analysis

The experiments were carried out using a completely randomized design, with three replicates. The data were subjected to analysis of variance using the Statistica software (StatSoft, version 7.1, StatSoft, Inc., Tulsa, StateOklahoma, USA), and the means were examined by least significant difference (LSD) at the 0.05 probability level. Simple linear correlations were also estimated among the phenolic compounds identified.

## Results

**Figure 2:**
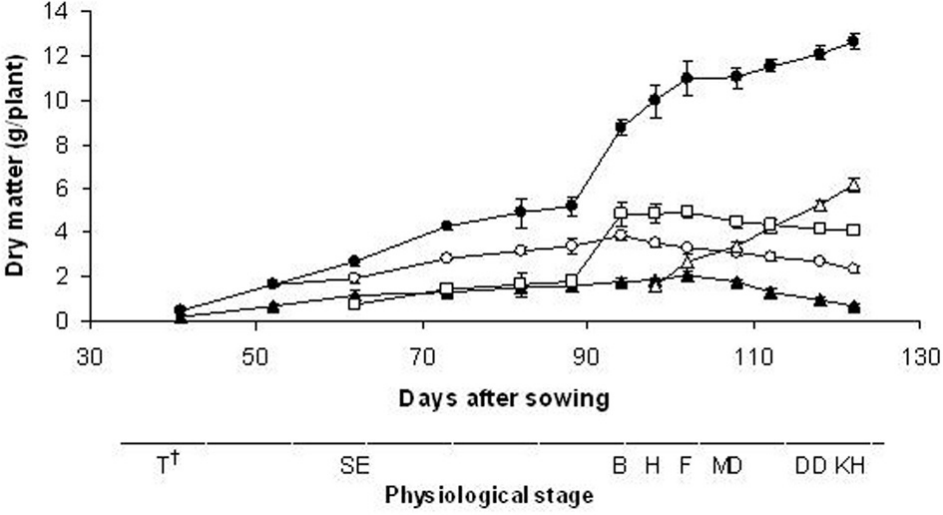
**Time-courses of dry matter accumulation patterns of total aerial biomass (•) and plant fractions (○, leaf; □, stem; Δ, ear; ▲, root).** Data are means of three replicates, vertical lines represent ±standard error. ^†^T, tillering; SE, stem elongation; B, booting; H, heading; F, flowering; MD, milk development; DD, dough development; KH, kernel hard.

The total aerial biomass (stems + leaves + ears) increased throughout the experimental period, and the maximum weight occurred at 122 DAS (12.6 g/plant) even if the increase in the plant DM was only 15% from the flowering stage (102 DAS; 10.95 g/plant) (Figure 2). A greater proportion of the biomass was partitioned into the leaves up to stem elongation (88 DAS), and into the stems up to the milky ripe stage (112 DAS); after this stage, the ears contributed more to the total aerial biomass. The ears contributed 49% to the total dry mass yield at seed maturity (kernel hard) stage. Root growth increased until 102 DAS (2.07 g/plant), which corresponded to the flowering stage, and then decreased. The duration of growth was only 122 days, and seed growth continued for approximately 20 days. The harvest index was calculated to determine the plant efficiency for seed yield under our experimental conditions: this was 43%.

**Figure 3:**
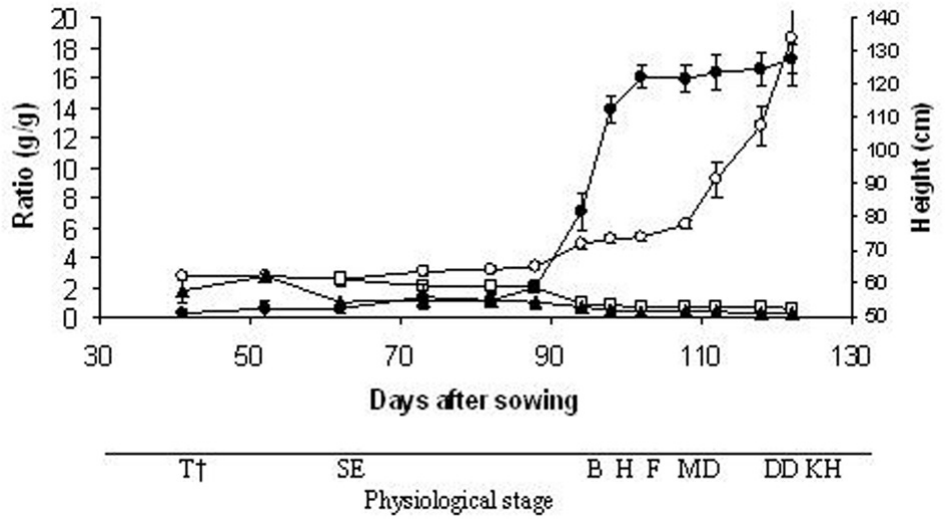
**Time-courses of plant height (•), leaf/ stem ratio (□), source/ sink ratio (▲) and shoot/ root ratio (○).** Data are means of three replicates, vertical lines represent ±standard error. ^†^T, tillering; SE, stem elongation; B, booting; H, heading; F, flowering; MD, milk development; DD, dough development; KH, kernel hard.

The plant heights reached 90% of the final value at the heading stage (98 DAS) (Figure 3). The LSR and SSR decreased progressively during the plant development; in particular, they both reached 65% of the initial value (2.49 and 2.18 g/g, respectively) at 94 DAS (booting stage). In contrast, the SRR at the last harvest (kernel hard stage) was 7-fold greater (18.58 g/g) than at the tillering stage. The RGR and SER calculated throughout the growth period were 36 mg g^−1^d^−1^ (*y* = 0.036 × −1.45; *r* = 0.95^**^; *n* = 13) and 1.19 cm d^−1^ (*y* = 1.19 × −17.67; *r* = 0.89^**^; *n* = 13), respectively. The mean RGR obtained with the classical approach was very similar (32 mg g^−1^d^−1^), however, considering the total growth period of 122 days, the RGR varied from 1 mg g^−1^d^−1^ at milk development stage (108 DAS) to 109 mg g^−1^d^−1^ at the tillering stage (52 DAS). A high RGR was also obtained at the booting stage (87 mg g^−1^d^−1^; 94 DAS). Linear regression analysis showing the allometric relationship between shoot and root dry weight revealed close correlation in the distribution of the dry weight between the shoots and roots throughout the growth period (*r* = 0.75^**^; *n* = 13). The slope of ln (shoot) vs. ln (root) was 1.11 (*s.e.* = 0.29), so the shoot/root ratios increased as the plants grew.

**Figure 4:**
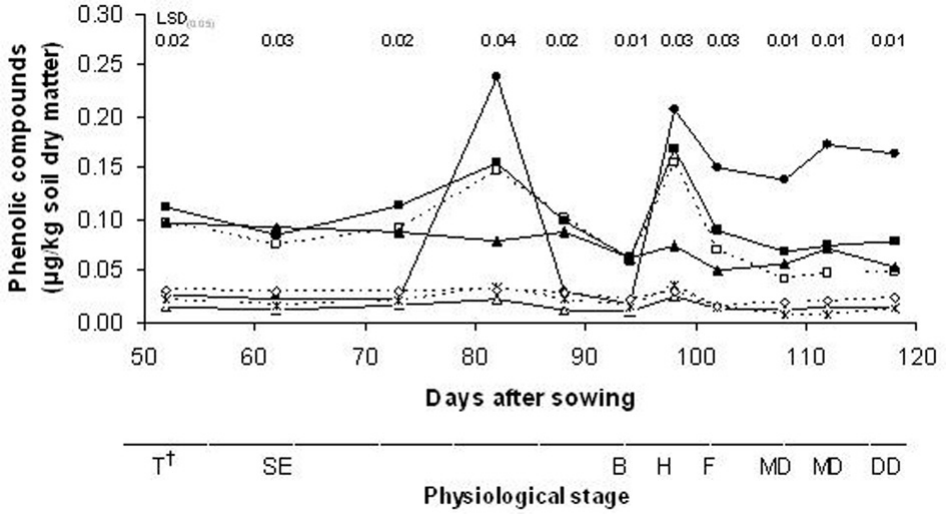
Time-courses of concentrations of phenolic compounds (—□, 4-hydroxybenzoic acid; —Δ, vanillic acid; —•, syringic acid; —■, vanillin; —*, *p*-cumaric acid; —▲, syringaldehyde; —◊, ferulic acid). ^†^T, tillering; SE, stem elongation; B, booting; H, heading; F, flowering; MD, milk development; DD, dough development; KH, kernel hard.

Analysis of the root exudates led to the identification of seven phenolic compounds in the rhizosphere soil from the wild oat roots: 4-hydroxybenzoic acid, vanillic acid, syringic acid, vanillin, *p*-cumaric acid, syringaldehyde and ferulic acid (Figure 4). The contents of these phenolic compounds in the rhizosphere soil of wild oat differed significantly across the stages of plant growth. All of these phenolic compounds identified in the rhizosphere soil except for syringic acid showed similar patterns during the growing season; in particular, there were two peaks that corresponded to the stem elongation (82 DAS) and heading (98 DAS) stages. Between these two peaks, the concentrations of the phenolic compounds in the rhizosphere soil declined suddenly. Later in the growing season, the phenolic contents decreased again.

As shown in Table 1, the mean levels of the phenolic compounds exuded into the rhizosphere soil by these wild oat plants during the growth cycle were ranked in decreasing order as: syringic acid, vanillin, 4-hydroxybenzoic acid, syringaldehyde, ferulic acid, *p*-cumaric acid and vanillic acid. The concentrations of the total identified phenolic compounds varied from 0.71 μg/kg at stem elongation (82 DAS) to 0.25 μg/kg at the booting stage (94 DAS). Syringaldehyde was the only compound that showed a maximum value in early plant development (tillering stage); the others reached their maximum concentrations at the stem elongation or heading stages.

**Table 1:** **Mean concentrations and ranges of the seven phenolic compounds identified in the rhizosphere soil of wild oat**. **The maximum values relating to the developmental stages of the plant growth are also given**.

**Compound**	**Concentration (μg/kg soil dry matter)**			**Growth stage of peak concentration**
	**Mean^†^**	**Range**	**Peak**	
4-Hydroxybenzoic acid	0.0855^b^^c^^†^	0.1134	0.1555^b^	Heading
Vanillic acid	0.0153^d^	0.0149	0.0251^d^	Heading
Syringic acid	0.1082^a^	0.2198	0.2382^a^	Stem elongation
Vanillin	0.1008^a^^b^	0.1053	0.1685^b^	Heading
*p*-Cumaric acid	0.0190^d^	0.0278	0.0354^d^	Heading
Syringaldehyde	0.0742^c^	0.0457	0.0971^c^	Tillering
Ferulic acid	0.0258^d^	0.0158	0.0313^d^	Stem elongation
LSD_(0.05)_	0.0186		0.0359	

† Means represent net concentrations (n = 33).

^‡^Values within a column not followed by the same letter are significantly different at P ≤ 0.05.

The letters

“a”, “b”, “c”, and “d” indicate that the values are statistically different.

Linear correlation coefficients were calculated to determine if there were any relationships between the seven phenolic compounds exuded from the roots of these wild oat plants during the plant growth (Table 2). Except for syringic acid and syringaldehyde, each of these compounds showed highly significant correlations with the other four compounds.

**Table 2:** **Correlation coefficients among the seven phenolic compounds identified in the rhizosphere soil of wild oat (*n* = 11)**.

**Phenolic compound**	**4-Hydroxy-benzoic acid**	**Vanillic acid**	**Syringic acid**	**Vanillin**	***p*-Cumaric acid**	**Syringaldehyde**	**Ferulic acid**
4-Hydroxybenzoic acid	1						
Vanillic acid	0.824^**^	1					
Syringic acid	0.273	0.651^*^	1				
Vanillin	0.964^**^	0.926^**^	0.415	1			
*p*-Cumaric acid	0.986^**^	0.814^**^	0.247	0.952^**^	1		
Syringaldehyde	0.471	0.161	−0.499	0.384	0.439	1	
Ferulic acid	0.713^*^	0.512	−0.194	0.670^*^	0.736^**^	0.835^**^	1

*, ** significant correlation at P ≤ 0.05 and ≤ 0.01, respectively.

## Discussion

The total aerial DM content of the wild oat increased linearly during the season. Therefore, despite the short duration of the growing period, a high biomass yield was obtained, probably because of the high value of the relative growth rate, particularly during the early period of plant growth. Moreover, a high value of the harvest index was recorded, even though the length of the reproductive period was only one sixth of the total plant growing period. Furthermore, the wild oat reached maximum root DM at the reproductive stage, and then declined to a minimum at the end of the growing season. Similar results were reported by El-Shatnawi and Makhadmeh (2002) in wild oat plants grown in pots. As reported by Barbour et al. (1998), the relative productivity and biomass of roots have an important adaptive significance in plants growing under arid and semi-arid conditions for the root physiological functions of absorption and translocation of water and nutrients, and synthesis of hormones and many other essential organic compounds.

The plants showed progressive decreases in LSR and SSR and an increase in SRR during the experimental period. This could be related to leaf senescence and/or the high demand for photosynthate during seed formation, which suggests that the sink strength can regulate the rate of photosynthesis (Calderini et al., 2006). As suggested by Olesen et al. (2004), some indicators of potential competitive abilities among crops and weeds can be obtained using plant growth analysis. Indeed, this approach quantifies the functional relationships that describe biomass accumulation and partitioning in a plant under non-limiting water, nutrient and light conditions. In particular, RGR (mass increase per unit time and mass) is a comprehensive trait of plants, which integrates the morphological and physiological characteristics, and is an important component of plant fitness (McBraw and Garbutt, 1990). The RGR maximum of the wild oat estimated here under our controlled conditions during the growing period was similar or greater to that reported by Villar et al. (2005) in 20 *Aegilops* species under field experiments. Furthermore, allometry, which measure biomass allocation among different plant organs, was used in the present study. These relations between time-dependent plant parameters have often been treated as genetically fixed characteristics of plant species or of groups of plant species (Niklas, 1995). As allometry is the quantitative relationship between growth and allocation, it can be used to predict the future performance with respect to biomass gain, or to analyse the adjustments to biomass distribution under contrasting environmental conditions. As shown by the allometric relationships calculated, the accumulation of shoot biomass is more highly correlated with the accumulation of root biomass in wild oat plants; moreover, a value for *b* greater than unity implies consistent bias toward shoot growth. As suggested by Bazzaz (1997), this probably means that in nutrient-rich environments, a small root system is sufficient for satisfying the nutrient requirements of the plant because the high nutrient availability compensates for the lower investment in root biomass.

Seven phenolic compounds: *p*-coumaric, ferulic, 4-hydroxybenzoic, vanillic and syringic acids, vanillin and syringaldehyde were identified in the rhizosphere soil of the wild oat. Both the analysis time for the determination of all of the phenolic acids (less than 30 min) and the profiles of the phenolic acids found are in agreement with those reported by others (Beato et al., 2011; Hernàndez et al., 2011). Unidentified peaks observed during HPLC analysis indicate that other phenolic compounds might be synthesized by wild oat. Many of the phenolic compounds found in the present experimental study have been found in root exudates of seedlings of wild oat (Pérez and Ormeno-Nuñez, 1991) and in other cereal species (Wu et al., 2001; He et al., 2006). Phenolic compounds have an aromatic ring that can bear one or more hydroxy substituents and derive from the shikimate pathway and phenylpropanoid metabolism. Phenolic acids consist of two subgroups, i.e., hydroxybenzoic and hydroxycinnamic acids; both benzoic and cinnamic acid derivatives have their biosynthetic origin in the aromatic amino acid L-phenylalanine. In many cases, aldehyde analogs are also grouped in with, and referred to as, phenolic acids (e.g., vanillin, syringaldehyde) (Li et al., 2010). Phenolic compounds have been the subjects of extensive research regarding their modes of action as plant-growth inhibitors. They have been documented to influence nutrient uptake, membrane permeability, protein synthesis, photosynthesis, respiration, enzyme activity, hormone balance and also water potential (Li et al., 2010).

The presence of phenolics detected in the soil inhabited by wild oat plants indicates that these might adversely affect the growth of other plants grown in this soil. Our analysis shows that the contents of these phenolic compounds in the rhizosphere soil of wild oat varies with plant age. Wu et al. (2001) noted that plant roots can regulate the exudation of allelochemicals into the growth environment to promote seedling allelopathy during the growing season. Furthermore, we found no relationship between the total DM accumulation and the quantity of phenolic compounds exuded into the rhizosphere soil of wild oat during plant development. As reported by Belz (2007), the biosynthesis and exudation of these metabolites follow a distinct temporal pattern and can be induced by biotic and abiotic factors. In particular, we recorded a fast decrease in their concentrations between the late stem elongation stage and the booting stage; over this same period, there was a high relative growth rate, because the plants were actively assimilating metabolites. As reported by (Poole, 2005), the inter-stage period of flag leaf fully emerged to ear emerging is characterized by intensive linear growth in cereals, whereby the crop canopy expands at its quickest and has the highest requirement for nitrogen. Furthermore, after the reproductive stage, the nutrient removal for the filling of the seeds becomes the most important biological process, which results in a decrease in the concentrations of many chemical compounds in the shoots, and presumably in the roots (El-Shatnawi et al., 2004). Therefore, in our study, these important biological processes were related to decreases in the concentrations of the phenolic compounds in the wild oat rhizosphere soil.

Our data show several associations among the phenolic compounds exuded. Indeed, correlation was found between the hydroxycinnamic acids measured (*p*-coumaric and ferulic acid). Highly significant correlations between the hydroxycinnamic acids and 4-hydroxybenzoic acid and vanillin were also observed. Similar results were obtained by Wu et al. (2001) in wheat root tissue; they concluded that accumulation of phenolic acids in roots could facilitate their exudation into the growth environment. Moreover, these correlations define the regression lines, which allow the determination of one phenolic acid concentration if the concentration of another is known. Finally, the present study showed that phenolic acid exudation from wild oat roots is a highly coordinated process over a wide range of plant sizes.

## Conclusions

The present study shows that wild oat roots can exude several phenolic compounds into their rhizosphere soil, and that the quantities exuded depend on each individual compound and on the developmental stage of the plant growth. This is important information for the management of crop–weed interactions in field crops, as the seriousness of the potential damage is also a function of the growth stage of the weed. However, the presence of phytotoxic compounds is not proof of an allelochemical function; further studies on their production, roles, and fates in the soil environment will be necessary.

### Conflict of interest statement

The authors declare that the research was conducted in the absence of any commercial or financial relationships that could be construed as a potential conflict of interest.
